# Report of the Section on Practice for the Second Congressional District

**Published:** 1883-06

**Authors:** T. S. Delke

**Affiliations:** Thomasville


### Societies.

---

### REPORT OF THE SECTION ON PRACTICE FOR THE SECOND CONGRESSIONAL DISTRICT.

By T. S. DELKE, M.D., of Thomasville.

In the following report taken from the Transactions of the Medical Association of Georgia, 1882, there are facts in sanitation, hygiene, and the history of certain types of disease, embracing a large and interesting region of the

State, that are too valuable to be hidden away in a limited edition of a volume of transactions:

As a member of the Committee of the Second Congressional District of Georgia on the Practice of Medicine, it behooves me to make some report on the same. After mature reflection and deliberation, I will—being prompted to endeavor to make a report on the prevailing diseases of this section, their type, character, etc, as well as the causes appertaining to them, as far as possible—thinking and taking the position that in this way alone and in analogous directions could a proper, consistent report—one that would meet the indication or the design of the committee—be made. I feel impressed that the dereliction of reports from committtes in this particular in the past, in a great measure, contributes to the lack of unanimity and innervation prevailing among the medical fraternity in the State, as well as the cause of the lethargy among the members of the Association. Reports in the past from committees, in a great measure, are not co-operative and general; but are mostly special reports, from individual practice, which might be considered a voluntary communication, and in fact should be so considered, as their value would be none the less estimable on that account.

Look to the teeming physicians of the country! Excite their observation. Stimulate them to interest in the great work that is being carried on; condense their observations into a report to the Association; the Association, by its critical test, eliminates the false, and arranges the facts for use—and the Association of Georgia, by its record of medical observation, will become an authority worthy of its mission.

Actuated, therefore, by the above impressions, I accordingly issued a circular to practicing physicians in the counties of Lowndes, Brooks, Thomas, Decatur, Mitchell and Dougherty. I recived replies from Drs. Burton and Rogers, of Lowndes; Dr. Patterson, of Brooks; Drs. Reid, Hopkins, Clower, Baston, Taylor, Bruce, McIntosh, Tullis, Culpepper and Mallette, of Thomas; Dr. Bruce, of Decatur; Drs. Cull and Twitty, of Mitchell; Dr. Alfriend, of Dougherty.

By reference to the map of the State, you will perceive that above named counties are in the extreme southern and western portion of the State, except Mitchell and Dougherty, which lie north of Thomas and Decatur, on the line of railway from Savannah via Thomasville, to Albany. The other four counties border on the Florida line, and are pierced by the S. F. & W. Railway.

The idea has prevailed in other sections of country north of this, that this section is a hot-bed of malaria. Now the reports received from the above named physicians, from the above named counties, go to show that fevers of malarious origin do not prevail to that extent that opinion from outside sources would suspect.

Taking in the first place, Drs. Burton and Rogers, of Valdosta, Lowndes county, to the

FIRST QUERY.

What class of diseases have been most prevalent in your practice the past year?

Answer.—No particular class of diseases prevail, except a few cases of typhoid fever, (to which I will in the future refer) and some diarrhœa, almost entirely confined to children in dentition.

2. Brooks.—No special class of diseases prevailed.

3. Thomas.—Diseases other than malarial prevailed most.

4. Decatur.—Mild typhoid fever.

5. Mitchell.—Epidemic and contagious diseases.

6. Dougherty.—Measles last spring, and early summer, with pneumonia; later, autumnal fevers.

SECOND QUERY.

Did malarial fevers prevail to the same extent as the preceding year?

1. Lowndes.—Malarial fevers prevailed in a few localities.

2. Brooks.—Malarial fevers did not prevail to the same extent as preceding year.

3. Thomas.—From eleven active practitioners, eight saw less malarial fever; three report more in their practice.

4. Decatur.—Malarial fever did not prevail to the extent as the preceding year.

5. Mitchell.—Malarial fevers have greatly diminished in the past few years in the number of cases.

6 Dougherty.—Not purely malarial as before.

THIRD QUERY.

What type of malarial fevers prevailed most?

1. Lowndes.—Intermittent fever prevailed most.

2. Brooks.—Remittent fever prevailed most.

3. Thomas.—Intermittent and remittent about equal.

4. Decatur.—Remittent.

5. Mitchell.—Did not state special type.

6. Dougherty.—Remittent, complicated with typhoid element.

FOURTH QUERY.

Were they more malignant?

1. Lowndes.—Not malignant.

2. Brooks.—Not more malignant.

3. Thomas.—Less malignant.

4. Decatur.—Less malignant.

5. Mitchell.—More mild.

6 Dougherty.—Not malignant, but had a tendency to be continued.

FIFTH QUERY.

Did typhoid fever prevail?

1. Lowndes.—Typhoid fever did exist, but not generally, and traces its cases to a certain colored individual, who was attending school, at Atlanta; came home on a visit, sick with typhoid fever, and from that case the disease spread, first through his father's family, then to those who had visited and assisted in nursing the family. The disease did not spread outside of an area of one mile.

2. Brooks.—No report.

3 Thomas.—The predominating report points to the prevalence of the disease, not as an endemic, but occurring in the practice of several practitioners. Several cases were imported, whilst other sporadic cases occurred —the cause and source were not well defined.

4. Decatur.—Prevailed, though other practitioners doubted its existence; yet, when typhoid treatment was instituted, the patients did well; generally mild.

5. Mitchell.—Opinion conflicts as to the prevalence of the disease—Dr. Cull denying, Dr. Twitty affirming—though, he says, the cases were by no means great; generally mild.

6. Dougherty.—No typhoid fever—Dr. Alfriend; pure type—Dr. Hilsman.

SIXTH QUERY.

With reference to observation of casuation.

1. Lowndes.—One case imported was the means of spreading the disease to those who lived in the same family with the case and to those who visited and attended upon it.

2. Brooks.—No special observation as to casuation.

3. Thomas.—Various observations, which are suggestive.

Drs. Reid and Culpepper observed that where malarious fevers prevailed most in the past, they did not exist to a greater extent than in localities where heretofore it did not exist.

Dr. Hopkins—That type of diseases have changed in a great measure in the last four years—where we have had intermittent we now have remittents.

Dr. Mallette—Families using water from the same well, had for several years suffered from malarial fever, every year, until an application six inches deep of charcoal for several feet in circumference around the well. Since said application, the fever has disappeared, and they have been exempt for the past four years.

Drs. Taylor and Bruce—That wells being very low on account of drought last year, concentrating the germs thereby making the water drank the vehicle for a greater quantity of specific germs of disease.

My observation has been attracted to several coincidences or causes. Out of the number I select two:

First. A certain place with good drainage; no ponds or lakes within two miles; the nearest water-course two miles; pine forest; sandy loam, with red clay foundation; exempt from malarial diseases. Upon deadening and clearing of the forest (about seventy-five acres) and breaking the soil immediate to and adjoining the residence and quarters for hands, whilst the timber was in the fermenting and dying state, and the land being broken, the white as well as the colored families, contracted intermittent fever, and for two years suffered. After the timber had all decayed and the land became well cultivated, the disease disappeared, and for the past eight years malarial diseases only occur occasionally, and confined to one individual at a time, leaving the question of cause to be settled only upon the supposition that some other place than home was the source of infection.

Another: A certain gentleman whose family, as well as laborers, suffered yearly with malarial diseases. In the immediate neighborhood, about one-quarter of a mile northwest from his house, was a large cypress forest—probably containing some fifty or sixty acres—covered from six inches to about four feet in water. He conceived the idea that this was the source of infection; whereupon he ditched this pond, and after the water ran off, and as the summer came on, his family, as well as laborers on his plantation, became affected, and finally prostrated, with a malignant type of malarial fever. Himself and two other members of his family died, as well as some of his laborers. Others, who lived from three quarters to one mile away, were affected with malaria to a greater extent than at any season before. After the swamp had dried off, and a season sufficiently dry occurred, fire being applied and the swamp thoroughly brunt out, the disease gradually began to abate, and finally disappeared, so far as prevailing generally. A case now and then occurs with individuals.

If we take the above two observations, we observe a remarkable difference as to source from which the specific agency emanated. In the first instance, we have a high well drained forest, when deadened, cleared and plowed, produced chills universally among the families adjacent to it; and after the timber had died and the land had become cultivated, the chills and fever disappeared, and for eight years only occassionally a sporadic case occurred. In the second instance, we find a pond or cypress swamp covered with water, entirely opposite the first, where, it was supposed, emanated the poisonous element that effected the inhabitants around it, though only occasionally. We gather force, make an attack by draining the water off; that arouses its ire, and it hurls back its venom, prostrating whole families—and some unto death. Whilst its energies are at its height, we effectually apply the purifier—the fire—and rob it of its sting.

With these remarks, I will close. If, perchance, they may conduce to throw some light into your honorable body, or stimulate others of our brethren to closer observation, and cause them to throw their mite into the Association, my object will be accomplished.

